# Fatigue Performance of Double-Layered Asphalt Concrete Beams Reinforced with New Type of Geocomposites

**DOI:** 10.3390/ma14092190

**Published:** 2021-04-24

**Authors:** Piotr Jaskula, Dawid Rys, Marcin Stienss, Cezary Szydlowski, Michał Golos, Jacek Kawalec

**Affiliations:** 1 Faculty of Civil and Environmental Engineering, Gdansk University of Technology, 80-233 Gdansk, Poland; pjask@pg.edu.pl (P.J.); marcin.stienss@pg.edu.pl (M.S.); cezary.szydlowski@pg.edu.pl (C.S.); 2 Tensar International Limited, Blackburn, Lancashire BB1 2QX, UK; MGolos@tensar.pl; 3 Faculty of Civil Engineering, Silesian University of Technology, 44-100 Gliwice, Poland; jacek.kawalec@polsl.pl; 4 Tensar International s.r.o., 737 01 Cesky Tesin, Czech Republic

**Keywords:** geogrid reinforcement, fatigue of asphalt pavements, four-point bending test, reflective cracking

## Abstract

The reinforcement of asphalt layers with geosynthetics has been used for several decades, but proper evaluation of the influence of these materials on pavement fatigue life is still a challenging task. The presented study investigates a novel approach to the reinforcement of asphalt layers using a new type of geogrid composite, in which square or hexagonal polypropylene stiff monolithic paving grid with integral junctions is bonded to polypropylene non-woven paving fabric. The laboratory fatigue tests were performed on large asphalt concrete beams reinforced with the new type of geocomposite. Unreinforced samples were used as reference. Test results were analysed in several aspects, including the standardised approach based on stiffness reduction, but also using energy dissipation. The effect of reinforcement on pavement fatigue life was also estimated. Based on the obtained final results of fatigue life calculations, it can be concluded that the evaluated geogrid composites have an evident positive effect on pavement performance and have a significant potential to extend the overall pavement life, especially in the case of hexagonal grid.

## 1. Introduction

### 1.1. Literature Review

Pavement reinforcement with geosynthetic materials is generally recognised as a cost-effective solution for pavement reconstruction. In spite of the fact that such applications of geosynthetics have been used for decades, there is still no widely accepted universal method of including geogrid reinforcement in pavement design. Usually, each geosynthetic material producer develops a proprietary design procedure, in most cases based on empirical observations. These can be obtained through (1) asphalt beam or slab laboratory tests, (2) small-scale wheel tracking tests or (3) full-scale testing and field trials [1]. Each of the listed methods has its advantages and disadvantages. In the case of small-scale laboratory testing using asphalt beams or slabs, the most unfavourable factor is that loading conditions, stress/strain states and compaction process differ considerably from those occurring in the field. Moreover, the preparation of (usually) double-layered samples with reinforcement between the layers generates additional problems in terms of scale, dimensions and equipment capabilities. On the other hand, the cost- and time-effectiveness of laboratory tests will always prevail in comparison with full-scale testing and field trials.

One of the first experiments of this type was conducted by Brown et al. [2,3], who used prismatic asphalt beams reinforced with polymeric geogrid that were subjected to cyclic loading. A notch with a depth of 5 mm was cut in the bottom of the beam to initiate the cracking process. Two positions of the reinforcement were investigated—11 mm and 22 mm above the lower surface of the beam, which was placed on a rubber support base. The obtained results indicated that it was possible to increase fatigue life by 10 times in the case where geogrid reinforcement was located in the lower position. Similar results were obtained by Lytton [4], who reported an increase in fatigue life by more than 10 times for reinforced asphalt beams, even if their thickness (76.2 mm) was less than the thickness of reference beams without reinforcement (101.6 mm). Saraf et al. [5] used large asphalt beams with the size of 610 × 76 × 76 mm that were placed on a flexible base made of rubber and subjected to half sine wave cycle loading at the frequency of 2 Hz. Two different types of reinforcing layer were used (fabric and woven grid), which were placed 25 mm above the base of the sample. The obtained results clearly indicated that samples with woven grid reinforcement performed better in terms of fatigue life than samples with fabric reinforcement, which still performed better than unreinforced reference beams. Test scheme in which asphalt beams were placed on a continuous flexible support (rubber pad) was also used by Chang et al. [6], who investigated fibre glass geogrids as reinforcement. In this case, the effectiveness of using fibre glass geogrid expressed by ratio of fatigue life of reinforced samples to fatigue life of reference (unreinforced) samples reached 1.5–2.5 for fibre glass geogrid, with tensile strength of 100 kN/m and from 5 to 9 for geogrid with tensile strength of 200 kN/m. Further experiments conducted by Brown et al. [7] and Sanders [8] used a modified test setup, in which two rubber spacers were placed between the lower surface of the test beam and the steel baseplate. In this case, the fatigue life of samples with polymer geogrid reinforcement was 2.2 times greater than for the control section, while steel geogrid was even more effective, with fatigue life of up to 3 times greater than for unreinforced beams. Similar research was conducted by Montestruque, who tested high-tenacity polyester geogrid incorporating an ultra-light nonwoven fabric and bituminous coating [9]. In this case, however, the cyclic load was applied not only in the bending mode, but also in the shear mode. The geogrid was placed directly over the end of the notch, which was cut to the depth of 75 mm. Three values of pre-crack openings were used—3 mm, 6 mm and 9 mm, for which the factors of effectiveness of 6.14, 4.6 and 5.11 were used, respectively. Loading of asphalt beam placed on a flexible base was also used by Sobhan et al. [10] and Obando et al. [11]. In later years, with the spread of the four-point bending fatigue test method with prismatic asphalt beams (4PB-PR), this scheme became widely used in evaluation of geogrid reinforcement. One of its first research applications was conducted in Italy (Grilli [12], Virgili et al. [13]), where a typical 4PB-PR test configuration was used to evaluate several different types of asphalt pavement reinforcement (glass fibre geogrid, polyester geogrid and geomembrane) and the solid mathematical interpretation of the results was introduced. The obtained values of fatigue life and calculated improvement factors indicated that the type of geogrid reinforcement had to be precisely chosen before use, since some materials would perform better as stress-absorbing layers to prevent reflective cracking, and others should be used to extend the fatigue life of the pavement structure. A similar approach was adopted by Arsenie et al. [14], who used the same 4PB-PR test configuration, but with much larger asphalt beams, almost twice in length, and under a different mode (controlled strain). For certain test conditions (three warp yarns within the width of the asphalt beam, criterion II—number of cycles to reach 80% of the initial stiffness), an average improvement factor of 1.68 was obtained. The results presented by Arsenie also indicated that the standard failure criterion used for the 4PB-PR test, which assumes fatigue life as the number of cycles to a 50% reduction in stiffness modulus, may not be valid for geogrid-reinforced specimens, because the positive effect of reinforcement is still active well beyond this moment. Significant influence of geogrid reinforcement on fatigue life increase was also confirmed by Ferrotti et al. [15] and Canestrari et al. [16], who used the 4PB-PR test, but with standard sample dimensions and controlled stress mode. In contrast, Zieliński used a slightly more simplified three-point bending (3PB-PR) test scheme with controlled stress mode [17] and conducted the test at two temperatures of +18 and −2 °C. According to his results, nonwoven polyester geofabric used as a reinforcing layer had almost no effect on fatigue life, while the influence of polyester geocomposite and glass grid geocomposite combined with polyester nonwoven geofabric was significant. However, even with these materials, the positive effects of reinforcement were visible only at the temperature of +18 °C. Ge et al. [18] investigated the behaviour of asphalt concrete beams reinforced with glass fibre-reinforced plastics (GFRP) using the 4PB-PR test and noticed that the application of such a reinforcing layer can extend fatigue life by almost 9 times, which would be especially useful for obtaining durable pavements on steel bridge decks. According to Zofka et al. [19], who conducted both 4PB-PR fatigue and monotonic 3PB tests using the specially developed advanced material characteriser, geogrid reinforcement is particularly effective in retarding crack propagation after the initial crack in the bottom layer has occurred. Chazallon et al. [20] and Arsenie et al. [21] presented a different approach in terms of sample preparation for the 4PB-PR test, in which three layers of asphalt mixture were separated by two layers of geogrid reinforcement, so that under sinusoidal loading, both the upper and lower zones of the sample were subjected to bending moment. With this setup, an average improvement factor of 1.5 was achieved for samples with geogrid reinforcement. In their 4PB-PR studies, Kumar and Saride [22,23] used double-layered reinforced and unreinforced samples with the bottom layer extracted from an old deteriorated pavement. Cracking propagation was recorded by means of a DIC system. Recently, the issue of fatigue life of geogrid-reinforced asphalt pavements was investigated by Orešković et al. [24,25] and Sudarsanan [26]. According to the literature review, geocomposite reinforcement can minimise crack propagation or significantly extend the fatigue life, but it may also reduce the inter-layer bond quality. Thus, the effect of geocomposite reinforcement on the extension of fatigue life of asphalt pavements is not obvious. Application of new types of geocomposites requires a more detailed investigation of their impact on inter-layer bond quality reduction and potential improvement of fatigue resistance of a multi-layered system.

Fatigue tests of various asphalt layer systems reinforced with geocomposites were conducted mostly in 4PB-PR and 3PB-PR test schemes. In several studies [27,28,29,30], a simple three-point bending test with monotonic load was also used, often to assess loading parameters for cyclic test [19]. The potential fatigue life of geogrid-reinforced pavement structures can also be investigated by means of wheel tracking test. Such experiments can be conducted using standard wheel tracking equipment [31,32] or with a custom-built station [33]. The fatigue test conditions are summarised in Table 1.

Based on the review of test conditions given in Table 1, the dimensions of the tested specimens typically do not exceed 400 × 100 × 100 mm and the maximum dimensions of the tested specimens in 4PB scheme equal 630 × 100 × 100 mm. Since the effect of scale may significantly influence the failure scheme of multilayered systems reinforced with geocomposites, it is advisable to perform laboratory tests on the largest possible specimens.

### 1.2. Aims and Scope

The main aim of the paper is to investigate whether reinforcement with the new type of hexagonal and square geogrid composites bonded with non-woven geotextile improves the fatigue resistance of asphalt layers, in the case when reinforcement slightly limits the full inter-layer bonding. For this purpose, laboratory tests of double-layered asphalt concrete systems reinforced with the new type of geocomposites were performed. Unreinforced samples were used as reference. Laboratory tests were performed on large specimens with dimensions of 850 × 170 × 100 mm, in order to minimise the scale effect. The tests delivered fatigue models (Woehler curves) and enabled the comparison of fatigue life of pavements reinforced with the new geocomposites and unreinforced pavements. The parameter ε_6_ obtained from this study will also aid practitioners in the design of geogrid-reinforced pavement structures.

## 2. Materials and Sample Preparation

### 2.1. Materials

#### 2.1.1. Asphalt Concrete

Tests were performed on double-layered specimens prepared in the following scheme:

- bottom layer made of asphalt concrete AC 11 W 35/50 for levelling course,
- upper layer made of asphalt concrete AC 16 W 35/50 for binder course.

Such a structural layout of pavement layers is widely used in Poland during pavement reconstruction. The AC 11 W asphalt concrete, which contains finer mineral mixture, fills cracks and potholes and provides overall smooth surface for the application of geogrid, which is later covered by binder course made of AC 16W asphalt concrete with coarser grading that provides general structural capacity of the reconstructed pavement.

Asphalt concretes were designed and produced at Road Research Laboratory, Gdansk University of Technology in accordance with the EN 13108-1 standard [34] and Polish technical guidelines WT-2:2014 [35] for medium traffic KR3-4 (from 0.5 × 10^6^ to 7.3 × 10^6^ of 100 kN standard axle loads, which corresponds to 1.2 × 10^6^ and 17.8 × 10^6^ of 80 kN standard axle loads), were used to fabricate specimens. The mineral mixture was composed of crushed gneiss/granite aggregate and mineral limestone filler.

The properties of bitumen used during the research program are shown in Table 2. All the properties were obtained by the authors on the basis of their own laboratory tests.

Table 3 presents the grading curve of both asphalt mixtures. Basic volumetric parameters are presented in Table 4.

#### 2.1.2. Geogrid Composites

**Figure 1:**
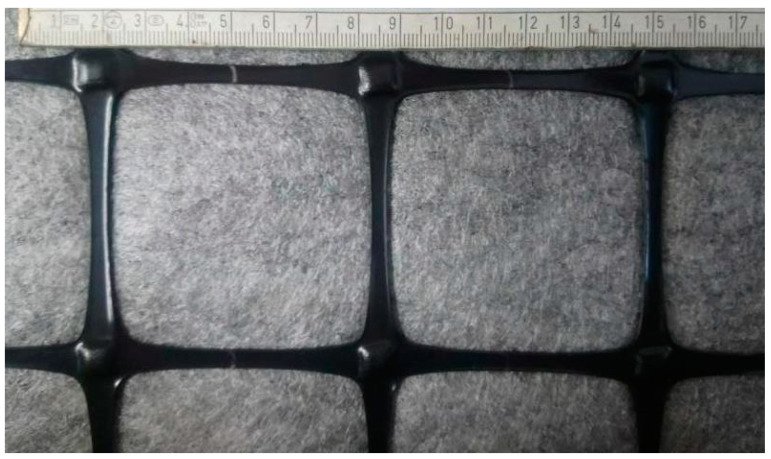
View of the AR-GN composite, square geogrid bonded to non-woven geotextile.

**Figure 2:**
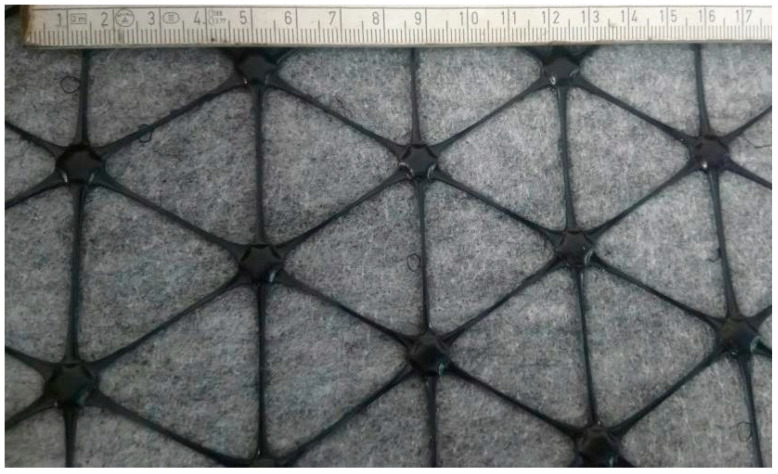
View of the AX5-GN composite, hexagonal geogrid bonded to non-woven geotextile.

Composites used in this research were produced by Tensar, Blackburn, Lancashire, UK. Two types of composite reinforcement were tested:

- composite interlayer AR-GN, i.e., square aperture geogrid bonded to non-woven fabric (Figure 1),
- composite interlayer AX5-GN, i.e., hexagonal geogrid bonded to non-woven fabric (Figure 2).

Both composites are structural paving composites consisting of two components: (1) a polypropylene stiff monolithic paving grid with integral junctions bonded to (2) a polypropylene non-woven paving fabric.

The grid is orientated in two directions in the case of square (biaxial) grid, or in three directions in the case of hexagonal grid. The distance between polypropylene strands of the square grid is 65 mm for both directions (see Figure 1), while the size of hexagon pattern of the hexagonal grid has the nominal value of 80 mm (see Figure 2). The grids used in the described composites perform the structural reinforcement function (R) of the asphalt interlayer. The tensile strength of the reinforcement was 20 × 20 kN/m for the square grid and 16 × 20 kN/m for the hexagonal grid, with a peak strain of approximately 11% in the longitudinal and transverse directions at maximum load, when tested in accordance with EN ISO 10319. The reinforcing elements are non-biodegradable and not susceptible to any chemical compounds that can occur in soils naturally or due to winter maintenance (e.g., aqueous solutions of salts). At least 2% of carbon black is added to the polypropylene during production process to ensure that the grid will not be susceptible to UV light during transport and storage.

The non-woven fabric used in both types of composites functions as a bonding layer during installation. Once installed and saturated with bitumen, it also acts as a SAMI layer and thus provides stress relief (STR) function. Another function provided by the non-woven fabric is moisture interlayer barrier (B). The described fabric has a residual bitumen retention of approximate 1.1 kg/m^2^ (square composite) and 1.5 kg/m^2^ (hexagonal composite).

Unit weight of the grid component is approximately 225 g/m^2^ (square) and 210 g/m^2^ (hexagonal), while the unit weight of the fabric component is 130 g/m^2^.

#### 2.1.3. Asphalt Emulsion for the Tack Coat

Based on the recommendation of the geogrid composite producer, the bitumen emulsion for surface treatments with designation C 69 B3 PU (Bitunova, Warsaw, Poland) was selected to be applied as a tack coat. The emulsion was produced from 100/150 neat bitumen, whose content equalled 69%. The residual bitumen amount after emulsion decay equalled 0.2 kg/m^2^ for unreinforced systems and 1.2 kg/m^2^ in the case of reinforced systems. Higher content of tack coat is necessary when composite is applied, to ensure proper inter-layer bonding and stress relief function. It has to be emphasised that introducing a composite between two asphalt layers causes partial loss of inter-layer bonding, despite a higher amount of tack coat. The direct shear strength test (Leutner test) according to [41], was used to assess the decrease in inter-layer bonding. Shear strength *τ*_max_ parameter is widely used in Poland for evaluation of inter-layer bond quality [42,43]. Average shear strength at +20 °C for systems without reinforcement equalled 1.26 MPa, while for the systems reinforced with hexagonal and square-aperture composites, it equalled 0.39 MPa and 0.32 MPa, respectively. According to studies presented by Jaskuła [43], even during emergency braking of a heavy vehicle, the calculated horizontal stresses at the depth of the interface between the binder course and the asphalt base course do not exceed the level of 0.15–0.20 MPa, which is almost twice as low as the inter-layer bonding strength obtained for samples with geogrid reinforcement.

### 2.2. Sample Preparation

The asphalt mixtures were prepared using a mechanical mixer according to the EN 12697-35 standard [44]. Before compaction, a short-term ageing process was conducted, during which loose portions of both mixtures were kept at 135 °C for 4 h, according to the procedure given in Appendix 2 to the WT-2:2014 document [35].

**Figure 3:**
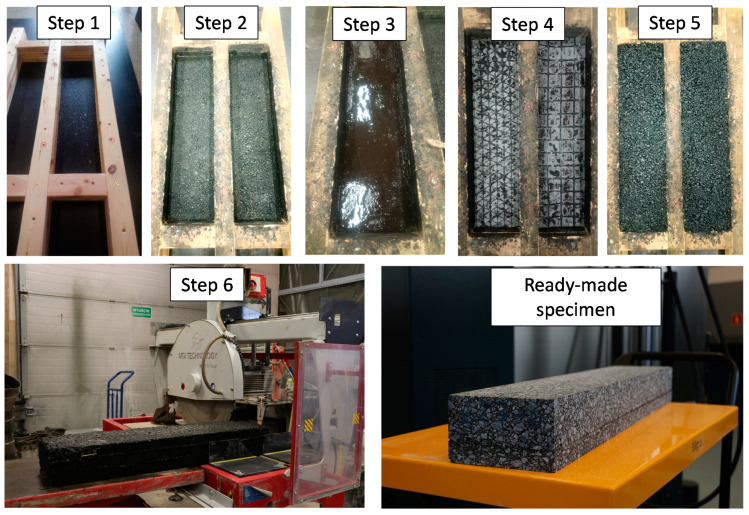
View of the successive steps of specimen fabrication. Step 1—completely assembled wooden mould. Step 2—compacted lower layer AC 11 W. Step 3—application of bitumen emulsion C 69 B3 PU. Step 4—application of geogrid composites. Step 5—compacted upper layer AC 16 W. Step 6—cut testing beam from compacted specimen.

Samples were produced using a specially prepared mould, enabling the formation of double-layered prismatic specimens. The mould consisted of a smooth, rigid base and a two-layered frame. The lower part of the frame, which enabled the compaction of the levelling course mixture AC 11 W, was 4.5 cm high. The upper part of the frame, enabling compaction of the binder course mixture AC 16 W over the previously laid levelling course, was 9.0 cm high. For samples with reinforcement, geogrid composite was laid on the surface of the previously compacted lower layer made of AC 11 W asphalt concrete, with proper tack coat application. Using the moulds, prismatic specimens were consecutively compacted to the dimensions of 250 × 135 × 1000 mm. They were afterwards sawn into target dimensions of testing samples of 170 × 100 × 850 mm, so that the final AC 11 W levelling course thickness of 30 mm and AC 16 W binder course thickness of 70 mm was obtained. The cutting process took place 14 days after compaction. The compaction procedure was designed to obtain the degree of compaction in the range from 98% to 100%. The mixtures were compacted using a single drum steel roller. All the surfaces were sawn in order to dispose of irregular surface areas of the sample that had been in contact with the side of the mould. Apart from prismatic samples dedicated to fatigue testing, cylindrical samples of 150 mm in diameter for inter-layer bonding tests were cored out from an additional set of prismatic specimens. The process of sample preparation is shown in Figure 3.

## 3. Methodology

### 3.1. Scheme and Course of the Inter-Layer Bonding Test

Inter-layer bonding was assessed using the direct shear test method, proposed by Leutner in 1979. It consists of the direct shearing (without bending) of layer interfaces in cylindrical asphalt specimens of 150 mm in diameter. Directly before the test, the specimens were conditioned for 12 h at the temperature of +20 °C. Shearing in the assumed failure plane progresses at the set displacement rate of 50 mm/min, up to the point of maximum shearing force and further, until complete shearing failure of the interface occurs.

The shearing force and the shearing displacement are measured during the test. The shearing strength of the interface *τ*_max_ is calculated from Equation (1) as the maximum registered shearing force divided by the area of the cross-section. After the maximum force is reached, the force gradually decreases with further displacement, which means that failure is initiated as the strength is reached, but complete failure of the interface does not occur instantly. Displacement values for each plot were corrected by fitting the tangent of the curve and determining the point where the tangent line meets the x-axis—thus obtaining the corrected initial displacement. Additional bonding assessment was based on shearing stiffness *k*, which is calculated on the basis of Equation (2).

(1) $$\tau_{max}=\frac{F_{max}}{A}$$

(2) $$k=\frac{\tau_{max}}{\delta_{max}}$$

where:

*F*_max_—maximum shearing force (MN),
*A*—area of the interface subjected to shearing (m^2^),
*τ*_max_—shearing strength (MPa),
*δ*_max_—corrected displacement at the moment of maximum stress (MPa),
*k*—shearing stiffness (MPa/mm),

### 3.2. Scheme and Course of the Fatigue Test

**Figure 4:**
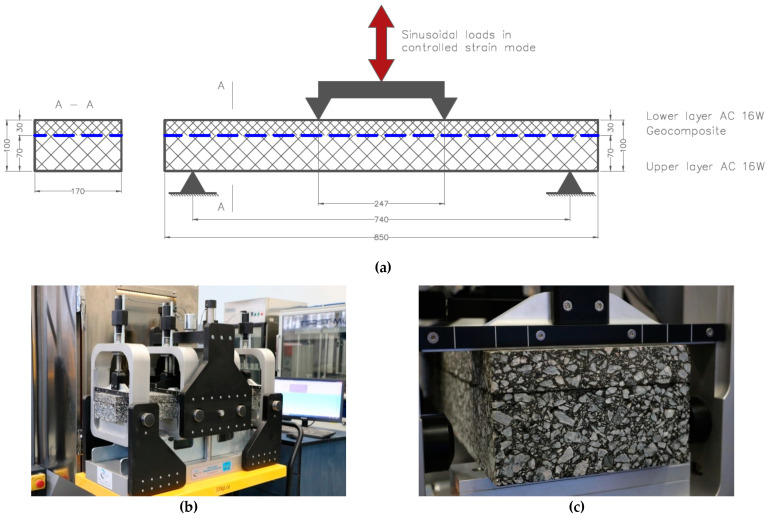
Four-point bending apparatus: (**a**) scheme of the 4PB test configurations (all dimensions in mm), (**b**) view of the test equipment outside the climatic chamber, (**c**) close-up of the beam cross-section.

Fatigue testing was performed in the scheme of four-point bending test (4PB-PR). A novel aspect of the approach was the use of significantly larger beams consisting of two layers of asphalt mixture with composite reinforcement and emulsion tack coat at their interface. Since such tests may be significantly affected by the scale effect, the specimens were larger than those used in typical procedures of fatigue testing with the 4PB-PR scheme. The adopted width of the sample (170 mm) was based on the assumption that the width of the geogrid placed between asphalt layers must cover at least 3 rows of geogrid strands. The distance between axes of the end supports equalled 740 mm. The distance between axes of the loading supports equalled 247 mm. Due to capabilities of the testing equipment, the specimens had to be placed in clamps of the testing machine in an inverted position in comparison to their natural arrangement in the pavement (i.e., the 30 mm of the lower AC 11W asphalt mixture and the reinforcement grid facing upwards). The loading actuator bent the beam upwards, so the location of the compression and tension zones within the specimen reflected real conditions. Such orientation of the specimen was assumed due to the character of the device and software used. The test was performed in a climatic chamber at a constant temperature of +13 °C, which is the equivalent temperature adopted in the design of flexible pavements in Poland. A view of the test equipment is presented in Figure 4.

**Figure 5:**
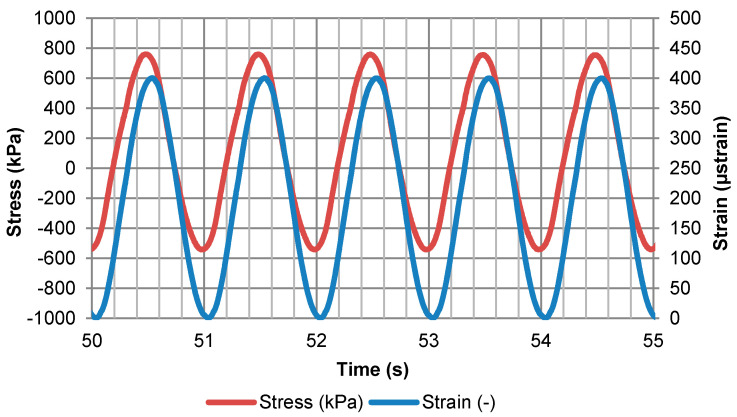
Example plot of stress and strain versus time/cycle number.

Beams were subjected to cyclic loading that caused a sinusoidal change in strain from 0 to the pre-set maximum value. The following maximum strain values were adopted: 400, 500, 600, 700 and 800 μstrain. For each strain level and for a given type of reinforcement, a single specimen was tested. This type of fatigue test is described in the literature as the “controlled strain mode”. Bending was forced only in one direction, so that the grid would function in the zone subjected to tension, like in a real pavement structure. The loading frequency equalled 1 Hz. An example plot of loading and strain versus cycle number is presented in Figure 5. The values given in Figure 5 represent the stress and strain calculated based on the measured loading forces, as well as displacement (deflection) in the middle of the beam. The maximum number of loading cycles equalled 300,000, which resulted in the maximum testing time of 84 h per test.

### 3.3. Methodology of Fatigue Life Evaluation

During the fatigue testing of asphalt mixtures in the controlled strain mode, the consecutive cycles result in a gradual decrease in the stiffness modulus (and, therefore, the stress induced in the specimen), which is indicated by the continuous decrease in the force needed to induce the adopted strain level. Typically, three stages of the stiffness modulus decrease are distinguished: (1) initial phase, which is characterised by a rapid decrease in stiffness, mostly due to effect of thixotropy, (2) phase of damage accumulation, when stiffness decreases gradually due to micro-crack formation within the asphalt mixture, (3) stage of failure, when stiffness decreases rapidly as an effect of macro-crack formation. The obtained results were analysed in terms of the initial stiffness, the number of cycles to failure and the total dissipated energy in the fatigue test. A detailed description of the parameters is given below.

Initial stiffness S_ini_—equivalent to the stiffness modulus of the system in the 100th loading cycle in the test. Stiffness in any given cycle may be calculated from (3). The parameter is not calculated in the first cycles due to the effects of thixotropy (a rapid decrease in stiffness under loading), as well as the stabilisation of the device and sensors that occurs during the first stage of the test. In the presented research, the initial stiffness was calculated using the data from the 100th cycle.

(3) $$S_{i}=\frac{\sigma_{i}}{\varepsilon }$$

where:

*S_i_*—stiffness in the *i*-th cycle of the test,
*σ_i_*—maximum tensile stress in the *i*-th cycle,
*ε*—maximum strain of the extreme tension fibres of the cross section, in the case of the controlled strain mode this value, is constant for the duration of the entire test.

Fatigue life *N_f_*—number of cycles to fatigue failure of the specimen. Different approaches are used regarding the limit of fatigue life. The most popular definition of fatigue failure is the moment at which the stiffness *S_i_* decreases to 50% of the initial stiffness *S_ini_* (from the 100th cycle of the test).

Another approach to the determination of fatigue life limit is the method proposed by Hopman [45], who defined the “Energy Ratio” (ER) term to evaluate failure process during fatigue test:

(4) $$ER=\frac{n*w_{0}}{w_{i}}$$

where:

*n*—number of load cycle,
*w*_0_—dissipated energy during the first cycle,
*w_i_*—dissipated energy during the *i*-th cycle.

Rowe modified and adopted in his works [46] the concept proposed earlier by Hopman. In his studies, the formula (4) was transformed in order to clearly identify the failure point. According to this method, the *R_ε_* parameter, defined for strain-controlled test by (5), is calculated for every load cycle of double-layered systems:

(5) $$R_{\varepsilon ,i}≅\frac{n}{S_{i}}$$

where:

*R_ε,__i_*—reduced energy ratio in the *i*-th cycle of the strain-controlled test,
*S_i_*—stiffness of the system in the *i*-th cycle,
*n*—number of cycles from the beginning of the test.

The point when the curve of the *R_ε_* parameter drawn on a graph as function of the number of cycles starts to deflect from the tangent line indicates the moment when asphalt mixture begins to develop macro-cracks. The number of load cycles in this moment may be considered as the fatigue life of the mixture.

Fatigue curve—determined by the *N_f_* fatigue life results obtained at various strain levels. The curve enables the evaluation of the system’s fatigue life at any given strain level, even outside of the tested range. The general form equation of the curve is as follows:

(6) $$\varepsilon =A{\left(N_{f}\right)}^{b}$$

where:

*ε*—tensile strain,
*A*—slope of the fatigue curve,
*b*—coefficient based on the obtained fatigue test results,
*N_f_*—fatigue life of the system.

Critical strain at one million load cycles ε_6_—parameter based on the fatigue curve, equivalent to the strain level at which the system’s fatigue life equals one million load cycles. This parameter is used for the characterisation of asphalt mixtures in terms of fatigue resistance. Higher ε_6_ strain level indicates better fatigue performance of the mixture (or the entire tested layered system).

**Figure 6:**
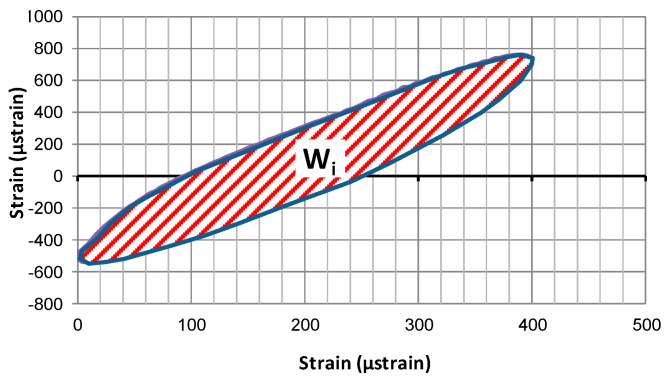
The area within the hysteresis loop in a given period (50th cycle) of the fatigue test, reflecting the energy dissipated in this cycle.

Dissipated energy in the given period (test cycle) *W_i_*—the value represents the energy (per unit of volume) spent on the formation of micro-cracks or transformed into heat. The dissipated energy may be interpreted as the area within the hysteresis loop—i.e., the curve obtained by plotting the relative changes of stress and strain during single loading cycle (shown in Figure 6). In a model case, when an elliptic shape of the hysteresis loop is assumed, the dissipated energy may be calculated from (7):

(7) $$W_{i}=\pi \bar{\varepsilon_{i}}\bar{\sigma_{i}}\sin\bar{\phi_{i}}$$

where:

*W_i_*—dissipated energy in the *i*-th test cycle,
$\bar{\varepsilon_{i},\;}\bar{\sigma_{i,\;}}\bar{,\phi_{i}}$—average —average amplitudes of strain, stress and phase angle, respectively, in the *i*-th cycle of the test.

Total dissipated energy from the start of the test to specimen failure *W_fat_*—the cumulative dissipated energy for all cycles from the start of the test to fatigue failure of the loaded system after *N_f_* cycles, expressed with (8):

(8) $$W_{fat}=\overset{N_{f}}{\underset{i=1}{\sum }}W_{i}$$

where:

*W_i_*—dissipated energy in the *i*-th cycle according to (7),
*i*—number of the cycle,
*N_f_*—fatigue life.

## 4. Results and Discussion

### 4.1. Direct Shear Testing

**Figure 7:**
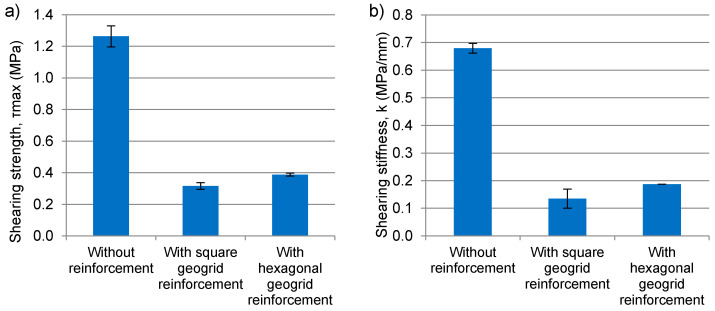
Shearing strength (**a**) and shearing stiffness (**b**) of systems without reinforcement, with hexagonal geogrid and with square aperture geogrid reinforcement.

The results of the direct shearing test are presented in Figure 7. Introduction of composite geogrid/geotextile reinforcement between the two layers of asphalt mixture resulted in a decrease in inter-layer bonding strength and shearing stiffness by 75% as compared to specimens without reinforcement. The type of composite interlayer used affects the shearing strength and stiffness. Use of composite reinforcement in the pavement will cause a reduction in the shearing strength and shearing stiffness of the inter-layer bond. It does not necessarily mean, however, that the pavement will be prone to the development of premature distress caused by the loss of inter-layer bonding. It is noteworthy that the shear strength of inter-layer bond is still greater than the maximum shear stresses, which equals approximately 0.3 MPa.

### 4.2. Flexural Stiffness of Double-Layered Systems with and Without Reinforcement

**Figure 8:**
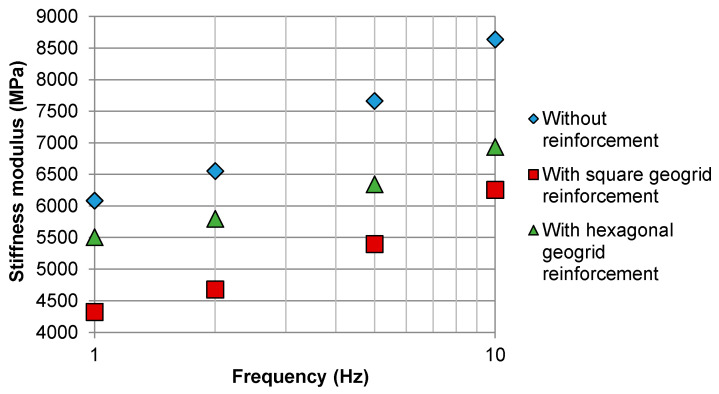
Comparison of the initial stiffness obtained at different load frequencies for double-layered asphalt concrete beams reinforced with square and hexagonal geogrids, as well as for reference samples without reinforcement.

Figure 8 presents the influence of test load frequency on the stiffness modulus of the systems. The analysis of frequency was performed at the controlled strain mode of 100 μstrain after 100 conditioning cycles. The low level of strain was chosen to minimise the accumulation of fatigue damage in specimens during the stiffness modulus test.

For both types of geogrid composites, the initial stiffness of reinforced specimens was lower by 13% to 26% in comparison with specimens without reinforcement. This behaviour was likely due to the presence of a relatively thick layer of bitumen-soaked fabric between the asphalt layers, which acted as a stress relief membrane and thus reduced the inter-layer bonding.

**Figure 9:**
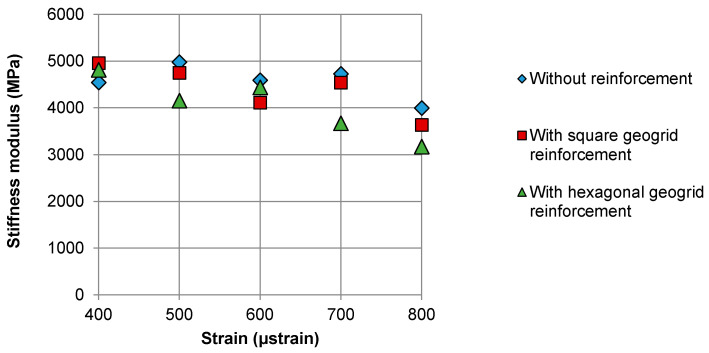
Comparison of the initial stiffness obtained at different strain values for double-layered asphalt beams: without reinforcement and reinforced with square and hexagonal geogrids.

Figure 9 shows the S_ini_ initial stiffness of the systems determined at various strain levels and with 1 Hz test frequency. Since the initial stiffness was measured during the 100th load cycle, its value decreases with the increase in the adopted test strain. It was noted that in most cases, the reinforced systems exhibited lower initial stiffness than the systems without reinforcement. In most cases, the stiffness was lower for systems with hexagonal geogrid than for those with square aperture geogrid. As previously described, lower initial stiffness of specimens with geogrid reinforcement was probably caused by the reduced inter-layer bonding between the lower and upper asphalt layer.

### 4.3. Fatigue Life Evaluation

**Figure 10:**
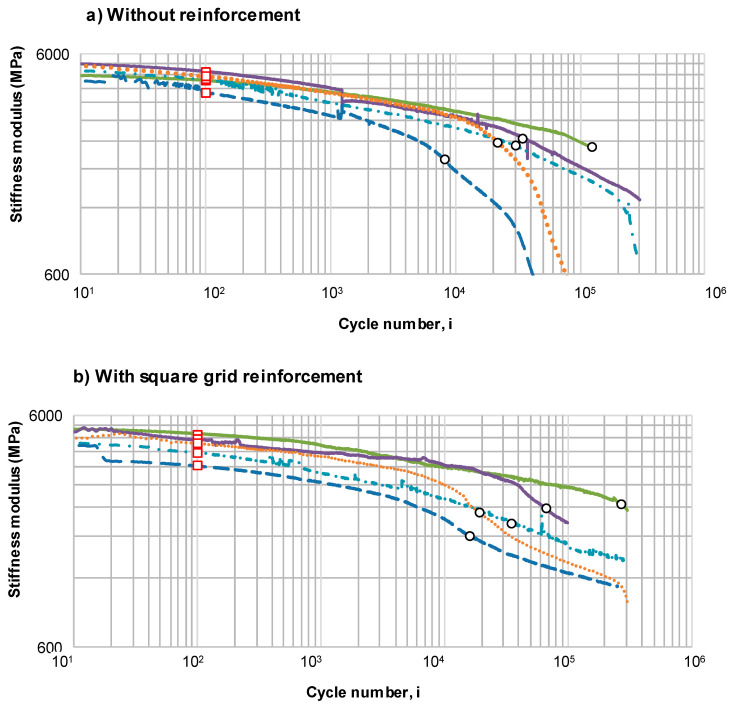

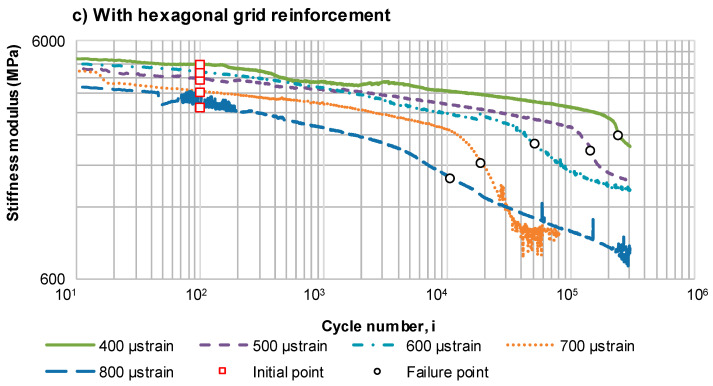
The change in stiffness modulus of double-layered asphalt beams: (**a**) without reinforcement, (**b**) with square grid reinforcement and (**c**) with hexagonal grid reinforcement.

During the fatigue test, the stiffness of the system gradually decreases. The rate of this decrease is shown in Figure 10, plotted separately for fatigue tests at different maximum strain levels: 400, 500, 600, 700 and 800 μstrain. Figure 10 also presents the values of initial stiffness and the points of fatigue failure, according to the criterion which defines failure as a decrease in stiffness to 50% of S_ini_.

The change of stiffness for specimens without reinforcement, shown in Figure 10a, is typical for the fatigue testing of asphalt mixtures. The points marked as “failure points” show the number of cycles at which stiffness modulus decreased to 50% of its initial value. Irrespectively of the variant that was tested (without or with reinforcement), a clear phase of gradual fatigue accumulation can be observed. While in the case of specimens subjected to strains of 400 and 500 μstrain, this phase lasts until the end of the test (300,000 cycles), in the case of specimens subjected to strains of 600, 700 and 800 μstrain, it develops into the third phase around the 200,000th, 15,000th and 1000th cycle, respectively. During the third phase of fatigue, a rapid decrease in stiffness occurs, which indicates the formation of macro-cracks in the asphalt mixture and may signal a significant weakening of the inter-layer bond as well. The stiffness modulus decreased to 50% of its initial value (marked as failure point) in the second phase for strains 400, 500 and 600 μstrain, and in the third phase in the case of strains of 700 and 800 μstrain. Failure points and points of transition from the second to the third stage are not equal.

In Figure 10b,c, plotted for specimens reinforced with composite interlayers, it is visible that the rate of decrease in stiffness in the 2nd phase is lower than for systems without reinforcement. Some of the reinforced specimens enter the 3rd phase of fatigue, but after a number of cycles, the curve inflects and its slope decreases again—this phenomenon is not observed for specimens without reinforcement. It indicates that at a certain level of fatigue the geogrid reinforcement activates to a greater degree, resulting in a notably less intensive decrease in stiffness modulus in further load cycles than in the case of systems without reinforcement. It is therefore expected that the development of fatigue cracks in pavements reinforced with composite interlayers will be slower than in the case of pavements without asphalt reinforcement.

**Figure 11:**
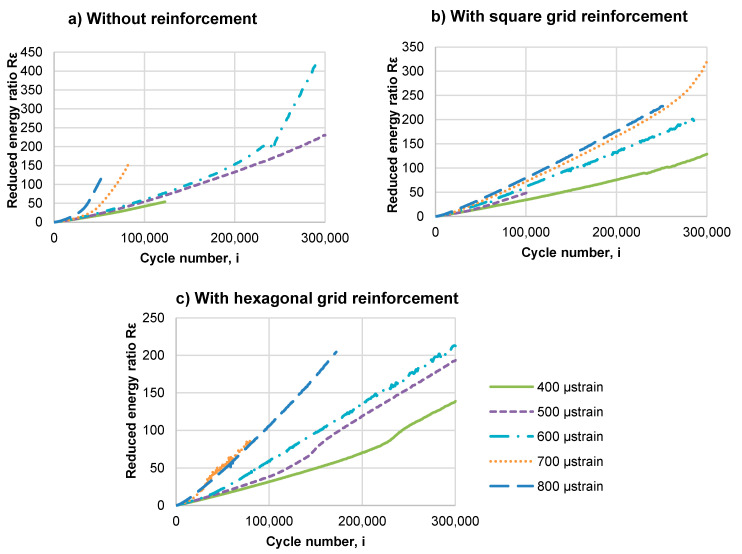
The change in the energy parameter R_ε_ of double-layered asphalt concrete beams: (**a**) without reinforcement, (**b**) with square grid reinforcement and (**c**) with hexagonal grid reinforcement.

Figure 11 shows the change in the R_ε_ energy ratio calculated using Equation (3). According to a theory presented by Rowe [46], the moment when the curve of the equivalent energy parameter R_ε_ increases above the tangent to the R_ε_–N plot indicates the asphalt mixture’s critical fatigue point, at which macro-cracks begin to form in the mix. However, visual observations of specimens after fatigue tests did not reveal the visible fracture of asphalt layers nor delamination of particular layers. It means that the loss of modulus was caused by accumulation of fatigue damage which was not exhibited in the form of visible cracks. In order to evaluate the impact of geogrid reinforcement on fracture resistance of the layered system, the specimens need to be notched.

In Figure 11, which presents the values of the R_ε_ reduced energy ratio calculated for specimens without reinforcement, the curves plotted for specimens subjected to strains of 600, 700 and 800 μstrain have evident deflection from the tangent line, after which the value of the R_ε_ parameter increases nonlinearly. For specimens subjected to strains of 400 and 500 μstrain, the deflection of the plotted function R_ε__(i)_ from the line tangent to the plot is not visible, which indicates that, according to the energy criterion, failure did not occur during the test (300,000 cycles). In the case of specimens reinforced with the grid, the plotted functions of R_ε__(i)_ are mostly linear till the last load cycle. The specimen reinforced with square grid subjected to 700 μstrain (see Figure 11b) is the exception, as it achieved failure at N_1_ = 268,000 cycles. In the case of specimens with hexagonal grid (see Figure 11c) subjected to 400 μstrain and 500 μstrain, after a nonlinear increase of the parameter (around 230,000 cycles and 130,000 cycles, respectively), the R_ε_ parameter began to increase linearly again, which means that the system did not undergo fatigue failure, but rather changed the mode of its functioning. According to the energy criterion, fatigue failure did not occur for the reinforced specimens, but it did occur for the specimens without reinforcement. It is a clear indication that the introduction of reinforcement has a positive effect on the fatigue life of double-layered systems. Nevertheless, it is impossible to determine the increase in the fatigue life of reinforced pavements solely on the basis of the energy criterion. Due to this fact, the criterion based on a decrease in stiffness by 50% of the initial value was employed as the limit of fatigue life in further analysis.

**Figure 12:**
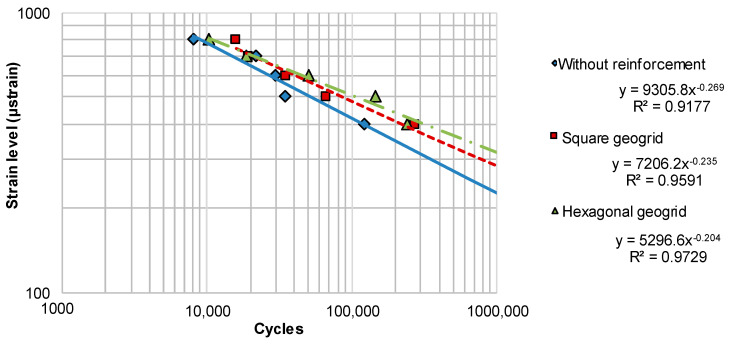
Fatigue model chart for double-layered asphalt beams.

Figure 12 shows the number of cycles to failure versus strain in the fatigue test. The marked points were determined on the basis of Figure 10 and represent the cycle numbers at which the stiffness decreased to 50% of S_ini_, constituting the limit of fatigue life. Based on the points representing the results of the performed tests, the fatigue model charts were plotted by fitting the lines to test data using the least squares method. The functions are presented in Figure 12.

**Figure 13:**
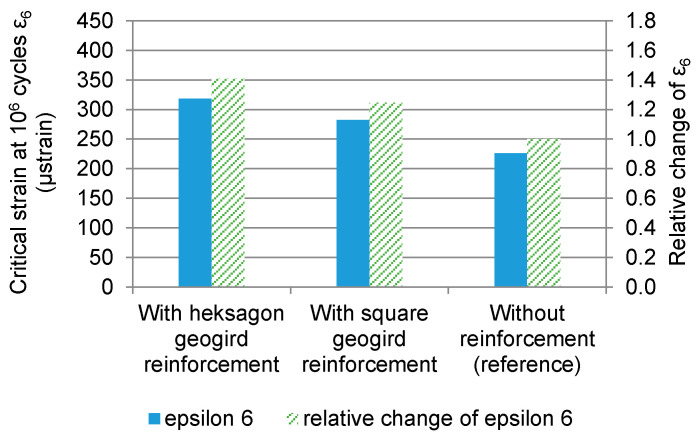
Comparison of critical strain ε_6_ and its relative change after introduction of reinforcement.

Based on the fatigue curves given in Figure 12, the critical strains at one million cycles ε_6_ were determined and presented in Figure 13. The ε_6_ parameter characterises the fatigue resistance of an asphalt mixture and is used in the French pavement design method developed by the LCPC institute [47]. Based on the ε_6_ critical strain, the systems were ranked according to their fatigue resistance. Specimens reinforced with hexagonal grid obtained the best result. Specimens reinforced with square grids ranked second. The lowest fatigue resistance was obtained for specimens without reinforcement. Figure 13 also presents the relative change in the ε_6_ parameter after the introduction of composite reinforcement. Reinforcement with square geogrid results in an increase in the critical strain by 25%, and with hexagonal geogrids—by 40%. For the sake of comparison, according to the French design method, a change from traditional asphalt concrete to high-modulus asphalt concrete results in an increase in ε_6_ by 24%.

### 4.4. Dissipated Energy

**Figure 14:**
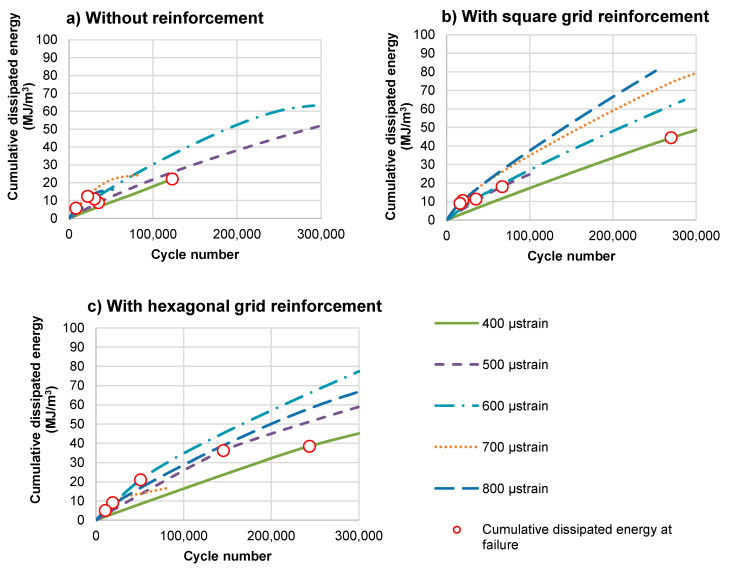
Accumulation of dissipated energy in consecutive test cycles for double-layered asphalt concrete beams: (**a**) without reinforcement, (**b**) with square grid reinforcement and (**c**) with hexagonal grid reinforcement.

Figure 14 presents the increase in cumulative dissipated energy of the double-layered beams, calculated from the start of the test. The marked points indicate the cumulative dissipated energy in moments at which particular specimens reached the fatigue limit according to the criterion of a 50% decrease in stiffness.

**Figure 15:**
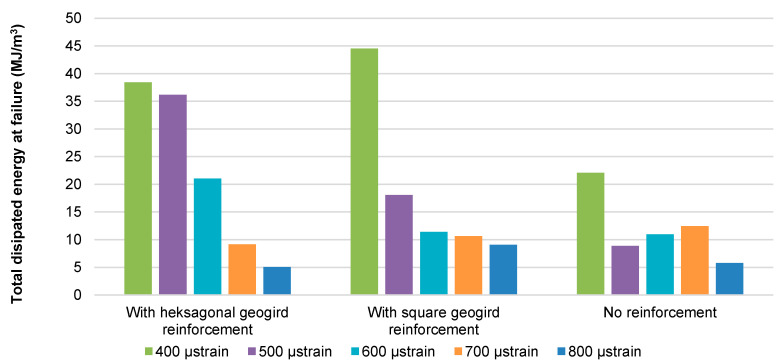
A comparison of the cumulative dissipated energy at failure (N_f_ load cycles).

Figure 15 shows a comparison of the total dissipated energy from the start of the test to fatigue failure defined as a decrease in system stiffness to 50% of the initial value.

As shown in Figure 15, at strain levels lower than 600 μstrain, the reinforced specimens dissipate significantly greater energy than the unreinforced specimens. It means that more energy is needed to cause the failure of a reinforced specimen. This effect is not evident for strain levels equal to or higher than 600 μstrain. Greater total dissipated energy at the moment of stiffness decrease by 50%, visible in Figure 14, reflects an improvement in the fatigue resistance of asphalt layers after the application of reinforcement.

### 4.5. Estimation of the Effect of Reinforcement on Pavement Fatigue Life

**Figure 16:**
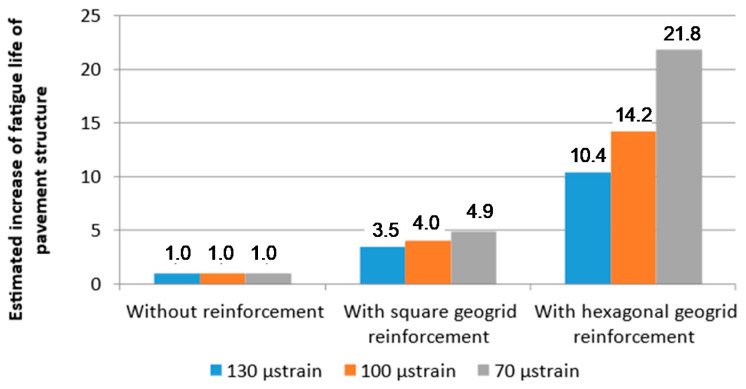
Estimated relative increase in fatigue life due to application of asphalt layer reinforcement at chosen levels of strain.

Based on the model fatigue charts shown in Figure 12, the estimated fatigue life values of the double-layered systems and entire pavement structures were evaluated. A strain of 130 μstrain was assumed, which typically occurs at the bottom of asphalt layers, under medium traffic in newly constructed pavements consisting of approx. 16 cm of asphalt layers placed on 20 cm of crushed aggregate base and subgrade with 100 MPa deformation modulus. The fatigue life of double-layered systems was estimated on the basis of Figure 12 and represents the number of cycles at 130 μstrain, leading to fatigue failure. Table 5 presents the fatigue lives of the considered double layered systems at 130 μstrain, as well as the relative increase in fatigue life obtained due to the introduction of reinforcement. The use of square aperture reinforcement resulted in an increase in fatigue life by a factor of 3.5, and the use of hexagonal reinforcement may even result in an increase by a factor of 10.4 in comparison to pavement without reinforcement. Figure 16 presents additional estimations of the relative increase in fatigue life for two different levels of strain at the bottom of the asphalt layers: 70 μstrain and 100 μstrain. The lower level of strain is representative of pavements with greater total thickness of asphalt layers. As shown in Figure 16, for lower levels of strain, the increase in fatigue life obtained through application of reinforcement is even greater. When analysing the presented results, the following factors should be taken into account:

- extrapolation of the fatigue model charts beyond the tested ranges,
- a decrease in overall stiffness of the asphalt layers due to introduction of reinforcement,
- possible different depth of the reinforcement within the pavement structure as well as potentially different proportions of thickness between the asphalt layers.

Nevertheless, the positive effect of composite interlayer reinforcement on fatigue life of the asphalt layers is evident.

The comparison presented in Figure 16 implies that the effect of asphalt reinforcement is particularly beneficial in the case of pavements for heavy traffic—characterised by the high overall thickness of the asphalt layers, deep placement of the reinforcement within the pavement structure and lower strains at the bottom of the asphalt layers. In the presented analysis, the effect of reinforcement in the case of pavements for light traffic (characterised by low overall thickness of the asphalt layers) will be limited, since the decisive criterion of failure for thin pavements is the permanent deformation criterion and not the fatigue criterion.

## 5. Conclusions

Based on the conducted research, the following findings may be formulated:

1. Introduction of geogrid reinforcement into specimens caused a decrease in their stiffness by approx. 20%. A decrease in direct shear strength by approximately 70% was observed as well.
2. A gradual decrease in stiffness modulus observed in the controlled strain fatigue test had a typical character in the case of specimens without reinforcement and an atypical character in the case of the reinforced specimens. An evident moment at which the geogrid activated during the fatigue test was observed.
3. All the tested specimens reached their fatigue limit according to the criterion that defines fatigue failure, as a decrease in stiffness by 50% of its initial value. According to the analysis of the reduced energy ratio R_t_, fatigue failure did not occur for the reinforced specimens, but it did occur for the specimens without reinforcement. It is a clear indication that the introduction of reinforcement has a positive effect on the fatigue life of double-layered systems.
4. The systems were ranked according to their fatigue resistance based on the critical strain ε_6_. The lowest fatigue resistance was obtained for specimens without reinforcement. Reinforcement with square geogrid resulted in an increase in the critical strain by 25%, and with hexagonal geogrid—by 40% (in comparison to system without reinforcement).
5. The results of fatigue tests were used for determination of fatigue models. Based on the fatigue models, the systems with hexagonal reinforcement achieved fatigue life from 10 to 22 times greater than systems without reinforcement; the systems with square grid reinforcement achieved fatigue life from 3 to 5 times greater than systems without reinforcement.
6. Based on fatigue life estimation analysis, it may be stated that geogrid reinforcement has an evident positive effect on fatigue life of pavements in the case of pavements for medium and heavy traffic loads.
7. Within the range of strains lower than 600 μstrain, specimens with square and hexagonal grid reinforcement dissipated more energy before failure than systems without reinforcement, which implies that pavements with reinforced asphalt layers would exhibit greater fatigue resistance.

## Figures and Tables

**Table 1:** Summary of fatigue test conditions based on literature review.

Literature	Test Scheme (Continuous Support, 3PB, 4PB, Mono, Cyclic)	Specimen Dimensions L × W × H (Lower + Upper) [mm], Notch [mm]	Type of Loading	Failure Criterion	Test Temperature/Frequency
Brown et al. 1985 [2,3]	Beam on continuous rubber base,	2-layered, 520 × 150 × 90 (11 + 79 or 22 + 68), notch of 5 mm	Cyclic load applied with steel and rubber plate	Propagation of crack 52 mm above the base	
Saraf et al. 1996 [5]	Beam on continuous rubber base	2-layered, 610 × 76 × 76 (22 + 51)	Cyclic load applied in the middle section of the sample	Total number of cycles to crack the entire thickness of beam	pulse 0.1 s, rest 0.4 s
Chang et al. 1999 [6]	Beam on non-continuous rubber base	2-layered, 508 × 76 × 76 (25 + 51)	Cyclic load applied in the middle section of the sample with steel plate	Total number of cycles to crack the entire thickness of the beam	30 °C pulse 0.1 s, rest 0.4 s
Brown et al. [7] Sanders [8], 2001	Beam on noncontinuous rubber base	2-layered, 420 × 200 × 90 (30 + 60), notch of 10 mm	Cyclic load applied with two metal clamps		
Montestruque et al. 2004 [9]	Beam on continuous rubber base	2-layered, 460 × 75 × 150 (75 + 75), width of the notch: 3 mm, 6 mm and 9 mm	Cyclic load applied with steel and rubber plate, bending or shear mode	Total number of cycles to crack the entire thickness of the upper layer	-/ 20 Hz
Sobhan et al. 2004 [10]	Beam on two separated pieces of plywood placed on continuous rubber base	2-layered, 457.2 × 152.4 × 76.2 (38.1 + 38.1) distance between the pieces of plywood: 10 mm	Cyclic load concentrated at the length of 12.7 mm in the middle section of the sample	Number of cycles to crack	2 Hz
Grilli, 2008 [12] Virgili et al. 2009 [13]	4PB, cyclic	2-layered, 305 × 100 × 75 (30 + 45)	Sinusoidal cyclic load, controlled stress mode F_max_ = 1 kN	Moment of failure calculated on the basis of the test curve, maximum number of cycles 20,000	20 °C 1 Hz
Arsenie et al. 2012 [14]	4PB, cyclic	2-layered, 620 × 100 × 90 (30 + 60)	Haversine cyclic load, controlled strain mode	Number of cycles at which 50% or 80% of the initial stiffness occurs	10 °C 25 Hz
Ferroti et al. 2012 [15] Canestrari et al. 2015 [16]	4PB, cyclic 3PB, quasi static	2-layered, 305 × 90 × 75 (30 + 45)	Haversine cyclic load, controlled stress mode force amplitude 1.0, 1.5 and 2.0 kN 50.8 mm/min	Flex point of the permanent deformation curve, maximum pre-cracking flexural load,	20 °C 1 Hz 20 °C
Montestruque et al. 2012 [31]	Beam in wheel tracking device on non-continuous PCC/rubber base	2-layered beams 40 mm HMA overlay and 20 mm of HMA stress relief layer,	Wheel tracking load	Total number of cycles to crack the entire thickness of the beam	-
Romeo et al. 2012 [27]	3PB, monotonic	2-layered beams 400 × 100 × 60 (20 + 40), plates 500 × 500 × 60 (20 + 40)	0.084 mm/s	Deflection of 40 mm	20 °C
Graziani et al. 2012 [28] Pasquini et al. 2013 [29]	3PB, monotonic	2-layered, 305 × 90 × 75 (30 + 45), 400 × 100 × 100 (50 + 50)	50.8 mm/min 5 mm/min	-	10 °C 20 °C
Zieliński, 2013 [17]	3PB, cyclic	2-layered, 300 × 75 × 75 (25 + 50)	Sinusoidal cyclic load, controlled stress mode, F_max_ = 2.5 kN or 5.5 kN	Number of cycles at which 50% of the initial stiffness occurs	−2 °C/+18 °C 10 Hz
Ogundipe et al. 2013 [32]	Beam in wheel tracking device on continuous rubber base	3-layered beams (with SAMI), 404 × 50 × 120 (30 + 10/20 or 30 + 80/70/60) notch depth = thickness of the bottom layer (30)	Wheel tracking load: 2.4 kN (1.1 MPa) or 1.35 kN (0.6 MPa)	Total number of cycles to crack the entire thickness of the beam	10 °C, 20 °C, 30 °C 0.8 Hz
Ge et al. 2014 [18]	4PB, cyclic	1-layered, 385 × 65 × 50	Haversine cyclic load, controlled stress mode	-	20 °C, 15 °C
Obando et al. 2015 [11]	Beam on continuous rubber base,	2-layered, 480 × 200 × 100 (50 + 50), width of notch: 4 mm, depth of notch: 16 mm	Cyclic load applied with 100 × 200 × 25 mm steel plate, controlled vertical stress mode	Total number of cycles to crack the entire thickness of the sample	-/ 1 Hz
Zofka et al. 2016 [19]	4PB, cyclic 3PB, monotonic	2-layered, 400 × 200 × 100 (30 + 70)	Sinusoidal cyclic load-controlled stress mode, force amplitude of 4 kN, 1 mm/min	Maximum number of cycles: 36,000	13 °C/1 Hz 13 °C
Chazallon et al. 2016 [20] Arsenie et al. 2017 [21]	4PB, cyclic	3-layered, 630 × 100 × 100 (25 + 50 + 25)	Sinusoidal cyclic load controlled strain mode	Maximum number of cycles 10^4^–2 × 10^6^	10 °C 25 Hz
Kumar and Saride, 2017 [22,23]	4PB, cyclic	2-layered, 400 × 50 × 90 (45 + 45), notch depth 25 mm or 40 mm	Haversine cyclic load controlled stress mode	Total number of cycles to crack the entire thickness of the sample	25 °C 1 Hz
Orešković et al. 2018, 2019 [24,25]	4PB, cyclic	2-layered, 400 × 60 × 50 (20 + 30)	Sinusoidal cyclic load Controlled stress mode	Number of cycles at which 50% of the initial stiffness occurs	20 °C 10 Hz
Vervecke et al. 2019 [30]	3PB, monotonic	2-layered (overlay on cement concrete), 580 × 180 × 85 (40 + 45)	0.4 mm/min 2 mm/min	-	4 °C −18 °C
Sudarsanan et al. 2020 [26]	4PB, cyclic	2-layered, 400 × 100 × 100 (30 + 70)	Haversine cyclic load Controlled stress mode	Moment of failure calculated on the basis of the test curve	10, 20, 30 °C 10 Hz

**Table 2:** Properties of 35/50 bitumen.

Property		35/50
Penetration at 25 °C (0.1 mm), acc. to PN-EN 1426 [36]	Original	45
	RTFO	28
R&B Temperature (°C), acc. to PN-EN 1427 [37]	Original	53.0
	RTFO	57.8
Performance Grade, acc. to AASHTO M 320		70–16

RTFO—Rolling Thin-Film Oven Test.

**Table 3:** Mineral gradation of the asphalt mixtures.

Sieve Size (mm)	%Passing (by Mass)	
	AC 11 W	AC 16 W
22.4		100
16	100	99
11.2	98	82
8	76	64
5.6	62	46
4	54	38
2	41	27
0.125	10	8
0.063	6.0	5.2

**Table 4:** Basic volumetric parameters of the asphalt mixtures.

Property	Test Method and Conditions	Obtained Value	
		AC 11 W	AC 16 W
Bitumen content (%)	-	4.8	4.5
Density, ρ_mv_ (Mg/m^3^)	EN 12697-5 [38], method A, in water	2.480	2.544
Bulk density, ρ_bssd_ (Mg/m^3^)	EN 12697-6 [39], method B, saturated surface dry	2.365	2.430
Air voids, V_m_ (%)	EN 12697-8 [40], Section 4	4.6	4.5
Voids filled with bitumen, VFB (%)	EN 12697-8 [40], Section 5	70.9	70.5
Voids in mineral aggregate, VMA (%)		15.8	15.2

**Table 5:** Comparison of the calculated fatigue life of pavements without reinforcement, as well as reinforced with square and hexagonal geogrid composites.

Type of Reinforcement	Strain at the Bottom of Asphalt Layers ε	Fatigue Life N_f_ (Laboratory Conditions)	Relative Increase in Fatigue Life
Without reinforcement (reference)	130	7,832,285	1.0
Square geogrid reinforcement	130	27,211,116	3.5
Hexagonal geogrid reinforcement	130	81,486,699	10.4

## Data Availability

Not applicable.
